# How housing burden damages residents’ health: evidence from Chinese cities

**DOI:** 10.3389/fpubh.2024.1345775

**Published:** 2024-05-20

**Authors:** Xiaoxin Guo, Shihu Zhong, Lin Li, Manyi Luo

**Affiliations:** ^1^Institute of Applied Economics, Shanghai Academy of Social Sciences, Shanghai, China; ^2^Shanghai National Accounting Institute, Shanghai, China; ^3^School of Business, Hunan University of Science and Technology, Xiangtan, China

**Keywords:** housing burden, physical health, psychological health, China, emerging economies

## Abstract

**Background:**

Currently, China is steadily pursuing high-quality development and promoting common prosperity, for which residents’ health is a precondition. However, high housing-price-to-income ratios and rent-to-income ratios have already triggered many social problems and have substantially affected people’s work and life. It is of practical significance to examine the relationship between housing burden and residents’ health.

**Methods:**

Combining city-level housing price-to-income ratio data and residents’ health data from the China Family Panel Studies, this study employs a binary logit model to investigate the impact and mechanism of housing burden on residents’ physical and psychological health.

**Results:**

Overall, a 1% increase in the housing-price-to-income ratio leads to a 1.2% decrease in physical health and a 1.9% decrease in psychological health. In terms of different psychological state indicators, a 1% increase in the housing price-to-income ratio leads to a 1.1% increase in depression, 1.1% increase in nervousness, 1.4% increase in relentlessness, 1.4% increase in hopelessness, 1.0% increase in a sense of incapability, and 1.4% increase in meaninglessness. According to mechanistic analyses, a 1% increase in the housing-price-to-income ratio leads to increases of 0.6 and 0.7% in the smoking rate and late sleep rate, respectively, while it leads to a 0.9% decrease in the noon nap rate.

**Conclusion:**

A growing housing burden significantly negatively impacts both the physical and psychological health of residents and increases the possibility of negative emotions. Further investigation revealed that the housing burden damages residents’ health by increasing their likelihood of smoking and sleeping late and decreasing their likelihood of taking a nap at noon, while exercise alleviates the negative impacts of the housing burden on residents’ physical and psychological health. Finally, we also find that housing burdens’ impacts on physical and psychological health differ significantly in terms of gender, age, and educational attainment. From the perspective of improving livelihoods, governments should consider the relationship between housing burdens and residents’ health when formulating livelihood policies. Location-specific and targeted policies should be followed. Additionally, efforts should be made to promote exercise among citizens.

## Introduction

Improving livelihoods and enhancing people’s sense of happiness constitute vital requirements to meet people’s desires for a better life, for which health is a key prerequisite. In other words, as the foundation for human activities, health strongly influences people’s lives. Therefore, health is cherished and aspired to by everyone (1). Moreover, health is also a vital part of human capital and is the fundamental factor for realizing economic development. Existing research has explored the impacts of different factors on health through the following perspectives: the impact of macrolevel factors on health, including migration (2, 3), urbanization (4), economic crises (5), environmental pollution (6), and housing price fluctuations (7); the impact of micro- and individual-level factors, such as gender (8), occupation (9), education (10, 11), and income (12), etc. However, only a small number of studies have examined the impact of housing on health. For example, Pollack et al. (13) explored the relationship between housing burden and diseases such as heart disease, diabetes, asthma, and psychological illness. However, no direct and statistically significant conclusion has been drawn, and it has been corroborated that housing burden negatively affects individuals’ health reports. Bentley et al. (14) and Mason et al. (15) reached similar conclusions based on data from different countries that the psychological health of low-income groups is significantly influenced by housing burden. Furthermore, Meltzer and Schwartz (16) tap into survey data of over 16,000 households in New York City and find that an increase in the self-paying housing burden leads to worsening health reports by individuals. Under such economic pressures, people may even delay medical treatment. Therefore, these studies serve as references and evidence for our study.

Housing is related to national development and people’s livelihoods, and price fluctuations affect the consumption decisions of residents to a large extent. Furthermore, it influences total social demand and has ripple effects on the macroeconomy. As the world’s largest developing country, China started market-oriented reform of housing in 1998. The real estate market boomed as housing prices increased. According to data from 35 large and medium-sized cities in China, the annual growth rate of average housing prices was approximately 17% from 2004 to 2013. However, during the same period, the growth rate of average income in these cities was approximately 11%, and the GDP growth rate in China was approximately 10% (17). Skyrocketing housing prices result in heavy housing burdens on residents. Housing, as the key factor affecting people’s subjective feelings of happiness and sense of benefits, has attracted the attention of various scholars. Studies have been conducted on the impact of housing-related factors on residents’ health, including housing quality (18, 19), housing conditions (20), and housing affordability (15). Currently, China is steadily pursuing high-quality development and promoting common prosperity, where residents’ health is a precondition. However, a high housing-price-to-income ratio has triggered many social problems and has substantially affected people’s work and life. It is of practical significance to examine the relationship between housing burden and residents’ health.

In response to practical needs from society and the research gap in the extant literature, this study employs data from the China Family Panel Studies (CFPS), housing prices, and income in prefecture-level cities and examines, following theoretical analysis, the impact of housing burdens on residents’ psychological and physical health in an empirical manner.

This study makes the following contributions. First, this study provides new evidence on the impact of the housing burden on the health of residents based on Chinese data. Existing research mainly uses samples from developed countries, which differ greatly from China in terms of housing market structure, housing price growth rate, building scale, homeowner vacancy rate, and government policy (21). China has different provinces with different levels of development and therefore regional disparities. The differences in housing prices in different areas due to regional disparities provide us with an opportunity to identify the impact of housing price-to-income ratios on the health of residents. Second, this study contributes to the investigation of the mechanisms through which housing burdens affect residents’ health. Existing research corroborates the negative correlation between housing burden and residents’ health. However, the impact mechanism has yet to be revealed to unravel the causal relation between housing burden and residents’ health. Third, this study investigates the heterogeneity of the impacts of housing burdens on different groups in a more detailed fashion. Through empirical analysis, we verify that the negative impact of the housing burden on residents’ health is particularly evident among certain groups, including females, older adult/adults people, and people with a high level of education. This offers evidence for targeted policy measures from governments.

## Theoretical analysis and hypothesis development

Housing burden is directly related to people’s livelihood and has been a focus of governments, the public, and academia. Housing burden refers to the housing cost residents have to pay to meet basic living needs (22). There is no consensus on the use of academics for measuring housing burden. Considering the data availability and practicability of the research method, scholars have adopted the housing-price-to-income ratio to estimate the housing burden (23, 24). Zhan et al. (25) estimated the housing-price-to-income ratio of 337 Chinese cities at and above the prefecture level in 2015 and 2019, respectively. They compare and contrast regional differences in the housing price-to-income ratio and find that the housing price-to-income ratio in China is generally high and is growing compared with the three-to-sixth range, which is considered reasonable globally. Therefore, in China, as the housing-price-to-income ratio increases, the housing burden of individuals and households also tends to increase.

The mismatch between skyrocketing housing prices and household income directly leads to an increase in residents’ housing burden, causing housing affordability issues for most households. Housing burden affects residents’ health through the following channels: first, households become less able to afford housing even with years of savings, which creates market anxiety and brings about considerable physiological pressure and direct economic pressure on residents; second, due to an increase in housing burden, residents reduce their sleeping time and time input in health and increase their working time and intensity in exchange for higher labor income to purchase housing or pay mortgages (26). As such, negative impacts on health are inevitable. Third, an increase in residents’ housing pressure directly influences their consumption decisions. Constrained by capital liquidity, people who plan to purchase housing or need to pay mortgages will shrink their spending in areas such as healthy food, health care, and health services (13), which ultimately damages their physical and psychological health. Fourth, an increase in the housing price-to-income ratio widens the income gap among residents via the wealth effect, resulting in a decrease in medical resource allocation and damage to residents’ health.

Health damage caused by housing burdens may trigger various negative emotions and adverse symptoms, such as emotional depression and insomnia. To alleviate such pressure and feelings of illness, individuals may resort to certain solutions, beneficial or detrimental, for comfort. Cases in point include exercising and smoking. It is certain that detrimental behaviors such as smoking exacerbate individuals’ physical and psychological damage. As a beneficial way to alleviate pressure, exercise helps maintain the dynamic equilibrium of our bodies, which prevents and cures chronic diseases of all sorts, enhances the human body, and improves the physical and psychological health of individuals (27). Moreover, exercise relieves pressure to some extent, eases depression, and improves individuals’ psychological health (28). However, it is worth noting that exercise should be limited within an appropriate range. Excess and improper methods of exercise increase the possibility of injury and harm personal health.

Based on the above analysis, we propose the following hypotheses.

*Hypothesis 1:* An increase in housing burden has a significantly negative impact on residents’ physical and psychological health.

*Hypothesis 1a:* An increase in housing burden pressure increases the likelihood of experiencing negative emotions such as depression.

*Hypothesis 1b:* 1b: An increase in the housing burden impacts residents’ health through bad habits such as sleeping late and smoking.

*Hypothesis 1c:* Exercise alleviates the health damage caused by housing burdens.

## Methods

### Data and variables

The data used in this study come from the 2010 China Family Panel Study (CFPS).^1^ The preliminary work for the CFPS survey began in 2007, with the questionnaire designed mainly by researchers from Peking University and with the participation of experts and scholars from many universities at home and abroad. Data collection began in 2008, with the presurvey conducted in 2008 and 2009 in Beijing, Shanghai and Guangdong, with a total of 2,400 households for initial and follow-up interviews; the formal survey opened in 2010, with a total of 1,986 samples distributed, and the final interviews were completed with 14,960 households, 33,600 adults and 8,990 children.

Given the large regional differences in Chinese society and to reduce the operational costs of the survey, the CFPS survey adopted an implicit stratification, multistage, multilevel population proportionate sampling (PPS) method to determine the target sample size. Among these variables, administrative division and socioeconomic level are the main stratification variables. Local GDP *per capita* is used as a ranking indicator of socioeconomic level within the same administrative division; where GDP indicators are not available, the share of nonagricultural population or population density is used as a proxy indicator.

The CFPS survey respondents cover a wide area, including a total of 25 provinces/municipalities/autonomous regions in China (excluding Hong Kong, Macau and Taiwan, as well as the Xinjiang Uygur Autonomous Region, Tibet Autonomous Region, Qinghai Province, Inner Mongolia Autonomous Region, Ningxia Hui Autonomous Region and Hainan Province) for both households and all family members in the sampled households. These 25 provinces account for 94.5% of the population of China as a whole, so the CFPS data can be considered a representative sample.

In terms of survey content, the survey focused on a number of research topics, including economic activities, access to education, family relationships and family dynamics, population migration, and the physical and mental health of Chinese residents, providing rich and representative data on basic individual- and family-level characteristics and mental health for this study.

In the process of data processing, because the most directly related person in a household is an adult individual and is more likely to be the head of the household, only the sample of the head of the household in each household was retained in the empirical analysis, and at the same time, the samples with missing values for the dependent variable, independent variable, and other key variables were deleted, resulting in the final total of 14,404 valid observation samples used in our study. The key variables are described as follows.

1. Independent variable. Housing burden (PIR) is the independent variable of this study and is measured by the housing price-to-income ratio of cities. Housing price data are derived from the sales value and sold area of commodity housing in the *China Statistical Yearbook for Regional Economy 2011.* The ratio of the sales value to the sales area of commodity housing denotes the average selling price (29). The income data are the *per capita* disposable income of urban residents in the *China Statistical Yearbook for Regional Economy 2011*.
2. Dependent variable. The dependent variable is individuals’ health conditions, which we measured through physical and psychological health. An individual’s health report, which is often considered an individual’s perception of their physical health condition, is adopted as a measurement of physical health. It is significantly correlated with objective health monitoring indicators such as disease and death risk (30) and therefore reflects individuals’ physical health conditions. As such, this study constructs the two-valued variable to denote individuals’ health reports based on the question “How do you like your health condition?” from the CFPS2010. The option “health” is assigned as 1, while the other options, including “just so”, “not so good”, “unhealthy”, and “very unhealthy”, are assigned as 0.

In terms of psychological health, the CFPS2010 employs the Center for Epidemiologic Studies Depression Scale, which comprises six scales that reflect major facets of depression. Each scale includes five options, i.e., nearly everyday, often, half of the time, sometimes, and never. In reference to Liu and Liu (7), this study constructed a depression scale score (CES-D) to measure individuals’ psychological health. The five options mentioned are assigned values of 0, 1, 2, 3, and 4. As such, the total score of the depression scale in this study falls into the range [0, 24]. The higher the score is, the fewer negative emotions the respondent has experienced over the previous month, and the better his or her psychological health condition. According to Burnam et al. (31), we take 4 as the critical point to construct a two-valued depression variable. A depression score higher than 4 is assigned a value of 1; otherwise, it is assigned a value of 0. In addition, to further analyze psychological health status, this study constructs subindicators of psychological health based on six scales, which include depression (Depress), nervousness (Nervous), restlessness (Restless), hopelessness (Hopeless), sens of incapability (Difficult), and meaninglessness (Meaningless). We construct two-valued variables of the six subindicators and assign them a value of 1 if the respondent chooses “nearly every”, “often”, “half of the time”, or “sometimes” and 0 if the respondent chooses “never”.

1. Control variable. Apart from key independent variables, we add a series of key control variables based on data characteristics and availability, including demographic statistics and socioeconomic characteristics. Control variables related to demographic characteristics include age (Age), gender (Female), hukou (Urban hukou), CPC membership (Communist), and marital status (Married). The socioeconomic control variables include higher education (College), secondary education (Middle and high school), and household income (Household income). The descriptive statistics and definitions of the variables are shown in Table 1.

**Table 1:** Summary statistics and variable definitions.

Variable	Mean	Std. dev.	Definition
Physical health	0.481	0.500	An indicator variable of people who report having a good physical health condition.
Psychological health	0.379	0.485	An indicator variable of people who have a good psychological health condition.
Psychology statuses			
*Depress*	0.469	0.499	An indicator variable of people who have ever felt depressed in the past month.
*Nervous*	0.368	0.482	An indicator variable of people who have ever felt nervous in the past month.
*Restless*	0.308	0.462	An indicator variable of people who have ever felt restless or fidgety in the past month.
*Hopeless*	0.219	0.413	An indicator variable of people who have ever felt hopeless in the past month.
*Difficult*	0.345	0.475	An indicator variable of people who have ever felt that everything was an effort in the past month.
*Meaningless*	0.196	0.397	An indicator variable of people who have ever felt life was meaningless in the past month.
PIR	16.73	29.83	The ration of the mean housing price at the district level to the household income.
Age	45.55	16.31	Age of individuals.
Female	0.520	0.500	An indicator variable of people being female.
Urban hukou	0.562	0.496	An indicator variable of people with an urban native hukou.
Communist	0.106	0.308	An indicator variable of members of the Communist Party of China.
Married	0.792	0.406	An indicator variable of people being married.
College	0.132	0.339	An indicator variable of people with a college degree or higher level of education.
Middle & high school	0.524	0.499	An indicator variable of people with middle & high school education.
Household income	4.479	4.190	Total household income, measured as 10,000 yuan/year.
Observations	14404		

Data source: China Family Panel Studies 2010.

### Econometric model

This study collected data based on an adult questionnaire from the CFPS2010 and the *China Statistical Yearbook for Regional Economy* to explore the impacts of housing prices on residents’ health. As residents’ physical and psychological health are two-valued variables, OLS regression may lead to heteroscedasticity, but the binary choice model better captures nonlinear impacts. Therefore, this study adopts a binary logit model for regression and constructs the following econometric model.


(1) $$Health_{ic}=\alpha_{0}+\alpha_{1}PIR_{c}+\alpha_{2}X_{ic}+\delta_{c}+\varepsilon_{ic}$$

where *i* and *c* represent the individual and region, respectively. $Health_{ic}$ is an individual’s health condition, which includes physical and psychological health; $PIR_{c}$ denotes the housing-price-to-income ratio at the city level. $X_{ic}$ is the group of control variables related to individuals’ health, including demographic and socioeconomic characteristics. $\delta_{c}$ is region fixed effects . $\varepsilon_{ic}$ is a random disturbance term. As this study uses cross-sectional data, there are no series-related issues.

## Results

### Benchmark results

Table 2 reports the impacts of housing burdens on residents’ physical and psychological health. For the sake of explanation, in addition to regression coefficients of the independent variable, we also display the marginal impacts of housing affordability on residents’ health conditions, or marginal effects. Train (32) noted that the average marginal effect is more reliable than the mean marginal effect. As such, this study reports average marginal effects. The regression results in Table 2 demonstrate that the housing burden exerts significantly negative impacts on both the physical and psychological health of residents, which are both statistically significant at the 1% level. A 1% increase in the housing-price-to-income ratio leads to a 1.2% decrease in physical health and a 1.9% decrease in psychological health. Thus, Hypothesis 1 is proved.

**Table 2:** Relationships between housing unaffordability and health conditions.

	(1)		(2)	
	Physical health		Psychological health	
	Coefficient	Marginal effect	Coefficient	Marginal effect
PIR (Housing-price-to income ratio)	−0.058***	−0.012***	−0.089***	−0.019***
	(0.022)	(0.005)	(0.021)	(0.004)
*Demographic characteristics*				
Age	−0.082***	−0.018***	−0.014*	−0.003*
	(0.007)	(0.002)	(0.007)	(0.002)
Age-squared	0.000***	0.000***	0.000***	0.000***
	(0.000)	(0.000)	(0.000)	(0.000)
Female	−0.347***	−0.075***	−0.251***	−0.054***
	(0.037)	(0.008)	(0.037)	(0.008)
Urban hukou	−0.173***	−0.037***	−0.003	−0.001
	(0.050)	(0.011)	(0.049)	(0.011)
Communist	0.142**	0.031**	0.194***	0.042***
	(0.064)	(0.014)	(0.068)	(0.015)
Married	0.031	0.007	0.513***	0.110***
	(0.056)	(0.012)	(0.055)	(0.012)
*Socioeconomic characteristics*				
College	0.093	0.020	0.027	0.006
	(0.074)	(0.016)	(0.074)	(0.016)
Middle & high school	0.084*	0.018*	0.281***	0.060***
	(0.047)	(0.010)	(0.047)	(0.010)
Household income	0.020***	0.004***	0.021***	0.005***
	(0.006)	(0.001)	(0.006)	(0.001)
*Unobserved regional characteristics*				
District fixed effect	Yes		Yes	
Pseudo *R*^2^	0.102		0.071	
Observations	14,404		14,404	

Robust standard errors in parentheses; ***p < 0.01, **p < 0.05, *p < 0.1.

To analyze the impact of housing burden on residents’ psychological health in an in-depth manner, we examine the effect of the housing-price-to-income ratio on different psychological states, including depression (Depress), nervousness (Nervous), restlessness (Restless), hopelessness (Hopeless), a sense of incapability (Difficult), and meaninglessness (Meaningless). The regression results are shown in Tables 3, 4. As the housing-price-to-income ratio increases, housing affordability decreases, and negative emotions such as depression, nervousness, restlessness, hopelessness, a sense of incapability, and meaninglessness significantly increase. Meanwhile, a 1% increase in the housing price-to-income ratio leads to the possibility of depression increasing by 1.1%, nervousness increasing by 1.1%, relentlessness increasing by 1.4%, hopelessness increasing by 1.4%, a sense of incapability increasing by 1.0%, and meaninglessness increasing by 1.4%. These results further demonstrate that increasing housing burdens impose significant negative impacts on residents’ psychological health.

**Table 3:** Relationships between housing unaffordability and different psychological states.

	(1)		(2)		(3)	
	Depress		Nervous		Restless	
	Coefficient	Coefficient	Coefficient	Marginal effect	Coefficient	Marginal effect
PIR	0.047** (0.020)	0.011** (0.005)	0.051** (0.021)	0.011** (0.004)	0.069*** (0.021)	0.014*** (0.004)
Demographic characteristics	Yes		Yes		Yes	
Socioeconomic characteristics	Yes		Yes		Yes	
Unobserved regional characteristics	Yes		Yes		Yes	
Pseudo *R*^2^	0.0749		0.0601		0.0594	
Observations	14,404		14,404		14,404	

**Table 4:** Relationships between housing unaffordability and different psychological states.

	(4)		(5)		(6)	
	Hopeless		Difficult		Meaningless	
	Coefficient	Marginal effect	Coefficient	Marginal effect	Coefficient	Marginal effect
PIR	0.086*** (0.023)	0.014*** (0.004)	0.047** (0.021)	0.010** (0.004)	0.096*** (0.023)	0.014*** (0.003)
Demographic characteristics	Yes		Yes		Yes	
Socioeconomic characteristics	Yes		Yes		Yes	
Unobserved regional characteristics	Yes		Yes		Yes	
Pseudo *R*^2^	0.0603		0.0678		0.0747	
Observations	14,404		14,404		14,404	

### Mechanism analysis

To unravel the mechanism through which housing burden affects residents’ health, this study constructs three two-valued variables—smoking (yes), noon napping (yes), and sleeping late (after 24:00)—based on the questions “Did you smoke over last month?”, “Do you take a nap at noon?”, and “When do you go to bed?” from the CFPS 2010 to examine the impact of the housing-price-to-income ratio on smoking, noon napping, and sleeping late.^2^ The results are shown in Table 5. We find that an increase in the housing price-to-income ratio positively and significantly affects smoking and sleeping late. In other words, an increase in the housing burden has a significantly positive impact on smoking and sleeping late. A 1% increase in the housing-price-to-income ratio causes the smoking rate and late sleep rate to increase by 0.6 and 0.7%, respectively. On the other hand, the housing price-to-income ratio has a significantly negative impact on noon naps. A 1% increase in the housing price-to-income ratio leads to a 0.9% decrease in the noon nap. These findings show that an increase in the housing burden damages residents’ physical and psychological health by increasing the possibility of smoking and sleeping late and decreasing the probability of noon naps. Therefore, Hypotheses 1a and 1b are justified.

**Table 5:** The effect of housing unaffordability on health conditions.

	(1)		(2)		(3)	
	Smoking		Noon nap		Sleep late	
	Coefficient	Marginal effect	Coefficient	Marginal effect	Coefficient	Marginal effect
PIR	0.045* (0.025)	0.006* (0.003)	−0.041* (0.022)	−0.009* (0.005)	0.100*** (0.033)	0.007*** (0.002)
*Demographic characteristics*						
Age	0.161*** (0.010)	0.020*** (0.001)	−0.023***	−0.005***	−0.027**	−0.002**
			(0.007)	(0.002)	(0.012)	(0.001)
Age-squared	−0.002*** (0.000)	−0.000*** (0.000)	0.000*** (0.000)	0.000*** (0.000)	0.000*** (0.000)	0.000*** (0.000)
Female	−3.734*** (0.144)	−0.465*** (0.009)	0.048 (0.037)	0.010 (0.008)	−0.422*** (0.067)	−0.030*** (0.005)
Urban hukou	−0.060 (0.072)	−0.007 (0.009)	−0.025 (0.049)	−0.005 (0.011)	−0.177** (0.086)	−0.013** (0.006)
Communist	−0.160* (0.089)	−0.020* (0.011)	0.317*** (0.065)	0.069*** (0.014)	−0.275** (0.122)	−0.020** (0.009)
Married	−0.040 (0.078)	−0.005 (0.010)	0.069 (0.056)	0.015 (0.012)	−0.351*** (0.100)	−0.025*** (0.007)
*Socioeconomic characteristics*						
College	−0.626*** (0.101)	−0.078*** (0.013)	0.310*** (0.073)	0.068*** (0.016)	−0.224* (0.128)	−0.016* (0.009)
Middle and high school	−0.340*** (0.062)	−0.042*** (0.008)	0.172*** (0.048)	0.038*** (0.010)	−0.168** (0.083)	−0.012** (0.006)
Household income	−0.002 (0.008)	−0.000 (0.001)	0.016***	0.003***	−0.001 (0.010)	−0.000 (0.001)
			(0.005)	(0.001)		
*Unobserved regional characteristics*						
District fixed effect	Yes		Yes		Yes	
Pseudo *R*^2^	0.3543		0.097		0.2071	
Observations	14,404		14,404		14,404	

It is universally accepted that an appropriate amount of exercise improves individuals’ physical and psychological health (33, p. 35). However, can exercise moderate the impact of the housing burden on residents’ health? Based on the answers to the questions ‘how many times did you exercise last week?’ and ‘how long do you exercise each time?’ in the CFPS 2010, we construct the two-valued variable of exercise. Samples that exercised at least three times and exceeded 30 min each time over the last week were assigned a value of 1; otherwise, they were assigned a value of 0. To investigate the moderating role of exercise, this study adds PIR * Exercise, the cross-product term between exercise and the housing-price-to-income ratio, for further regression, the results of which are shown in Table 6. We find that exercise affects residents’ physical and psychological health positively and significantly, which is consistent with our expectations. The regression results for PIR * Exercise show that PIR * Exercise is not significant in Model (3) but is significantly positive in Model (4). This demonstrates that exercise relieves, to some extent, the psychological burden caused by the housing burden. However, its effects on easing physical harm are not obvious. Thus, Hypothesis 1c is corroborated.

**Table 6:** Moderating effect of exercise on the correlation between housing unaffordability and health conditions.

	(1)		(2)		(3)		(4)	
	Physical health		Psychological health		Physical health		Psychological health	
	Coefficient	Marginal effect	Coefficient	Marginal effect	Coefficient	Marginal effect	Coefficient	Marginal effect
PIR	−0.057*** (0.022)	−0.012*** (0.005)	−0.088*** (0.021)	−0.019*** (0.004)	−0.068** (0.027)	−0.015** (0.006)	−0.112*** (0.025)	−0.024*** (0.005)
Exercise	0.140*** (0.041)	0.030*** (0.009)	0.172*** (0.041)	0.037*** (0.009)	0.140*** (0.041)	0.030*** (0.009)	0.170*** (0.041)	0.036*** (0.009)
PIR * Exercise					0.029 (0.038)	0.006 (0.008)	0.063* (0.036)	0.014* (0.008)
Demographic characteristics	Yes		Yes		Yes		Yes	
Socioeconomic characteristics	Yes		Yes		Yes		Yes	
Unobserved regional characteristics	Yes		Yes		Yes		Yes	
Pseudo *R*^2^	0.1026		0.0719		0.1026		0.0721	
Observations	14,404		14,404		14,404		14,404	

### Heterogeneity analysis

The impacts of housing burdens on residents’ health may vary among different groups. Using the questionnaire results of the CFPS 2010, this study divided the samples into different groups based on gender, age, and educational attainment for heterogeneity tests. The specific analysis is presented below.

### Heterogeneity effects according to gender

Due to differences in physiology, social status, and health-related behavior, the health conditions of males and females vary (34, 35). To further explore whether there is gender-based heterogeneity in the impacts of housing burdens on health, this study divides the sample into female and male groups for regression, the results of which are shown in Table 7. We find that an increase in housing burden significantly and negatively affects the physical health of females, while its impact on males is not significant. A 1% increase in the housing-price-to-income ratio leads to 2.2% growth in physical health damage among females. A possible explanation is that the conventional belief that males serve as breadwinners and that females take charge of domestic chores becomes obsolete as society progresses and pressure increases. Females shoulder more responsibility for housing costs. Compared with males, females have weaker physical strength and are therefore more vulnerable to physical damage caused by the housing burden. However, there is no evident heterogeneity in the impact of the housing burden on the psychological health of males and females. A 1% increase in the housing-price-to-income ratio leads to 2.1 and 1.6% growth in the psychological damage of females and males, respectively.

**Table 7:** The correlation between housing unaffordability and health conditions across gender groups.

	(1)		(2)	
	Physical health		Psychological health	
	Coefficient	Marginal effect	Coefficient	Marginal effect
PIR * Female	−0.103*** (0.030)	−0.022*** (0.007)	−0.100*** (0.027)	−0.021*** (0.006)
PIR * Male	−0.016 (0.028)	−0.003 (0.006)	−0.076*** (0.028)	−0.016*** (0.006)
*Other variables controlled*				
Demographic characteristics	Yes		Yes	
Socioeconomic characteristics	Yes		Yes	
Unobserved regional characteristics	Yes		Yes	
Pseudo *R*^2^	0.1022		0.0603	
Observations	14,404		14,404	

### Heterogeneity effects according to age

Age has a vital influence on individuals’ health. In addition, individuals differ greatly in terms of life situation and work status among various age groups. This study takes 30 years of age as a critical age and divides the sample into two groups, those aged 30 and above and those aged younger than 30, to investigate the heterogeneous impacts of housing burden on these two age groups. As the results in Table 8 show, an increase in housing burden negatively and significantly affects the physical and psychological health of people aged 30 and above, while having a nonsignificant impact on the health of people aged less than 30 years. A 1% increase in the housing-price-to-income ratio contributes to 1.4 and 1.9% growth in the physical and psychological health of people aged 30 and above, respectively. The possible reason is that people younger than 30 years are at the starting phase of their career. Their salary is relatively low. Even though they have demands in housing, their affordability is low. Therefore, their housing burden is often transferred to their parents. People at and above 30 years of age need to not only establish themselves but also shoulder the housing burden from the next generation. High housing prices exacerbate their housing burden, making them work longer and harder, decreasing their health and sleeping time, and ultimately wreaking havoc on their health (36).

**Table 8:** The correlation between housing unaffordability and health conditions across age groups.

	(1)		(2)	
	Physical health		Psychological health	
	Coefficient	Marginal effect	Coefficient	Marginal effect
PIR * Aged above 30	−0.063*** (0.023)	−0.014*** (0.005)	−0.090*** (0.021)	−0.019*** (0.005)
PIR * Aged below 30	−0.017 (0.056)	−0.004 (0.012)	−0.082 (0.056)	−0.018 (0.012)
*Other variables controlled*				
Demographic characteristics	Yes		Yes	
Socioeconomic characteristics	Yes		Yes	
Unobserved regional characteristics	Yes		Yes	
Pseudo *R*^2^	0.1020		0.0604	
Observations	14,404		14,404	

### Heterogeneity effects in educational attainment

Educational attainment is an essential factor influencing individuals’ income level and is also the key factor impacting their affordability in housing. To investigate education-based heterogeneity, this study adopts undergraduate education as the criterion and divides samples into two groups, people with schooling at or above undergraduate education (College) and people with schooling below undergraduate education (Noncollege). The results in Table 9 demonstrate that the housing burden has a significantly negative impact on the physical and psychological health of people who are educated at or above the undergraduate level. However, its impact on people with schooling below the undergraduate level is not statistically significant. A 1% increase in the housing-price-to-income ratio brings about approximately 1.3 and 2.1% growth in the physical and psychological health of people with schooling at or above the undergraduate level, respectively. A possible explanation may be that individuals with higher educational attainment enjoy competitive edges in the labor market compared with those with low educational attainment. They are more attracted to urban living philosophy and have a stronger intention to settle in cities. Health damage caused by the ‘housing slave effect’ is more likely to occur for people with higher educational attainment.

**Table 9:** The correlation between housing unaffordability and health conditions across education groups.

	(1)		(2)	
	Physical health		Psychological health	
	Coefficient	Marginal effect	Coefficient	Marginal effect
PIR * College	−0.062*** (0.022)	−0.013*** (0.005)	−0.099*** (0.021)	−0.021*** (0.005)
PIR * Noncollege	0.002(0.071)	0.000 (0.015)	0.082 (0.066)	0.018 (0.014)
*Other variables controlled*				
Demographic characteristics	Yes		Yes	
Socioeconomic characteristics	Yes		Yes	
Unobserved regional characteristics	Yes		Yes	
Pseudo *R*^2^	0.1020		0.0605	
Observations	14,404		14,404	

## Discussion and conclusion

This study matches the housing price data of Chinese cities with data from the China Family Panel Studies in 2010 and constructs a logit model to examine the impacts of housing burdens on residents’ physical and psychological health. First, we find that housing burdens exert significantly negative impacts on both physical and psychological health. Second, housing burdens significantly increase the generation of negative emotions such as depression, nervousness, restlessness, hopelessness, a sense of incapability, and meaninglessness. Third, the housing burden increases the likelihood of smoking and sleeping late, which is lower than that associated with noon naps by residents. Fourth, exercise alleviates the psychological damage caused by the housing burden, while it has little effect on alleviating the physical damage caused by the housing burden. Finally, the negative impact of the housing burden on the physical and mental health of women, people aged 30 and over, and people with a bachelor’s degree or higher is more pronounced.

The above findings reinforce and build on research on the relationship between the housing cost burden and residents’ health. Existing studies based on research contexts in different countries have revealed that the housing cost burden exacerbates residents’ negative emotions (37), leading to poor mental health and physiological health and significantly increasing their mortality rate (38), and has a significant negative impact on residents’ subjective health perception (39). However, there are limitations in the findings of these studies; for example, researchers have paid more attention to the health effects of housing burdens on older adult/adults people and children (40–42), but little attention has been given to the behaviors and health status of the main groups who actually bear the burden of paying for housing. This paper shows that the negative impact of housing burden on the health status of the group aged 30 and over is more significant and clarifies that housing burden has a significant impact on a range of health-related behaviors, such as smoking, sleeping late and exercising, further elucidating the pathways through which housing burden affects the health of residents. At the same time, we have made some discoveries in the discussion of gender and educational heterogeneity that provide new directions for future research.

The conclusions of this study have several policy implications. First, from the perspective of improving livelihoods, governments should consider the relationship between housing burdens and residents’ health while formulating livelihood policies. Tighter regulations should be imposed on the real estate market by deepening the reform of the housing security system to form a housing policy system covering different levels of and differentiated demands. Policies related to housing security and to curbing real estate speculation should be implemented consistently to avoid the negative impacts of skyrocketing housing prices on residents’ health. Second, efforts should be made to promote exercise among citizens. Exercise can ease, to some extent, the health damage caused by the housing burden. The government should increase its input in improving citizens’ health by implementing the Healthy China Strategy and National Fitness Program. By building a high-level public service system for fitness and improving the urban environment, the government can promote targeted support for residents’ health. Third, location-specific and targeted policies should be followed. Health damage from housing burdens is more common among females, young people, and well-educated people. For females, the government should improve the relevant supervision system to eradicate gender discrimination in the labor market, secure equal employment rights for females, and guarantee women’s rights and interests in property across the board; for low-income young people, the solution lies in affordable housing projects. The government should establish a rental housing market featuring diverse supply and multiple supportive channels to realize organic equilibrium between employment and housing. For well-educated groups, the government should offer housing subsidies and grant higher tax cuts or subsidies for purchasing housing. In addition, efforts should be made to improve the supervision mechanism to avoid subsidy deception.

The data used in this paper are not sufficiently recent due to limitations in data availability, and in the future, we can further supplement and refine this study if updated and representative household-level housing and health survey data become available.

## Data availability statement

Publicly available datasets were analyzed in this study. This data can be found here: https://opendata.pku.edu.cn/dataset.xhtml?persistentId=doi:10.18170/DVN/45LCSO. The raw data supporting the conclusions of this article will be made available by the authors, without undue reservation.

## Author contributions

XG: Conceptualization, Data curation, Formal analysis, Methodology, Software, Writing – review & editing. SZ: Writing – review & editing, Methodology, Funding acquisition, Conceptualization. LL: Writing – review & editing, Supervision, Project administration, Methodology. ML: Writing – original draft, Visualization, Project administration, Investigation, Formal analysis, Data curation.

## Footnotes

^1^Data access links: https://opendata.pku.edu.cn/dataverse/cfps?q=&types=datasets&sort=datesort&order=desc&page=1

^2^Since the mechanism analysis variables in this paper are all 0–1 dummy variables, it is more difficult to use structural equation model and it also does not address the problem of multiple dependent variables interacting with each other. Although structural equation model can be used to analyze the dummy variable data, the technical treatment is quite different because the dummy variable data cannot be analyzed on the basis of ordinary covariance matrices but only rely on covariance matrices based on multinomial correlation coefficients. While structural equation model can be applied to situations where multiple dependent variables are handled at the same time, in path analyses of structural equation model, even if multiple dependent variables are displayed in the graphs of the statistical results, the regression coefficients or path coefficients are still single computed for each of the dependent variables. As a result, structural equation model graphs may appear to consider multiple dependent variables simultaneously, but the presence and influence of other dependent variables is also ignored when calculating the effect or relationship of one dependent variable.
